# Proximate Mediators of Microvascular Dysfunction at the Blood-Brain Barrier: Neuroinflammatory Pathways to Neurodegeneration

**DOI:** 10.1155/2017/1549194

**Published:** 2017-08-14

**Authors:** Barry W. Festoff, Ravi K. Sajja, Luca Cucullo

**Affiliations:** ^1^PHLOGISTIX LLC, 4220 Shawnee Mission Parkway, Fairway, KS 66205, USA; ^2^Department of Neurology, University of Kansas Medical Center, 3901 Rainbow Blvd, Kansas City, KS 66160, USA; ^3^Department of Pharmaceutical Sciences, Texas Tech University Health Sciences Center, 1300 S. Coulter Street, Amarillo, TX 79106, USA

## Abstract

Current projections are that by 2050 the numbers of people aged 65 and older with* Alzheimer's disease* (AD) in the US may increase threefold while* dementia* is projected to double every 20 years reaching ~115 million by 2050. AD is clinically characterized by progressive dementia and neuropathologically by neuronal and synapse loss, accumulation of amyloid plaques, and neurofibrillary tangles (NFTs) in specific brain regions. The preclinical or presymptomatic stage of AD-related brain changes may begin over 20 years before symptoms occur,* making development of noninvasive biomarkers essential*. Distinct from neuroimaging and cerebrospinal fluid biomarkers, plasma or serum* biomarkers* can be analyzed to assess (i) the presence/absence of AD, (ii) the risk of developing AD, (iii) the progression of AD, or (iv) AD response to treatment. No unifying theory fully explains the neurodegenerative brain lesions but* neuroinflammation* (a lethal stressor for healthy neurons) is universally present. Current consensus is that the earlier the diagnosis, the better the chance to develop treatments that influence disease progression. In this article we provide a detailed review and analysis of the role of the blood-brain barrier (BBB) and damage-associated molecular patterns (DAMPs) as well as coagulation molecules in the onset and progression of these neurodegenerative disorders.

## 1. Introduction

### 1.1. Innate Immune Activation in CNS and Neurodegeneration

Often described as a double-edged sword [1, 2] or Janus-faced [3, 4],* neuroinflammation* is a host defense system for prompt recovery from various acute conditions in the CNS, both infectious and sterile [5–7]. In these situations, it is usually short-lived, accomplishing its task and setting the stage for repair and recovery. However, if prolonged and chronic it may also play detrimental roles leading to neurodegenerative processes. The innate immune system, in simple terms, consists of both pro- and anti-inflammatory “factors” and in the CNS responds to genetic influences, protein aggregates and abnormal cell constituents, injury-released mediators from neurons, and mechanism suppression that would otherwise control neuroinflammation. Systemic infection or injury causes an inflammatory response that transmits information to the brain, directing a metabolic and behavioral cascade known as “sickness behavior” [8]. As mentioned, in the brain this innate immune activation is short-lived, prompt, and well-organized but may be prolonged in sepsis or polytrauma that results in the systemic inflammatory response syndrome (SIRS) [9, 10]. With infection, pathogen-associated molecular patterns, such as the prototype endotoxin or lipopolysaccharide (LPS), bring about activation of surface pattern recognition receptors (PRRs) on immune and other cells for a robust inflammatory response mediated primarily via the Toll-like receptor (TLR) family, of which thirteen are now known but only eleven in humans [6, 11, 12].

The principal cells of the innate immune system, circulating monocytes or macrophages, collectively peripheral immune white blood cells (piWBC), exist in various phenotypes beyond the “classically activated” M1 (*γ*-interferon-exposed) or “alternatively activated” M2 macrophages following interleukin-4 or interleukin-13 treatment [14]. Similar evidence exists for microglia within the CNS [15]. Numerous publications and reviews have identified positive and negative roles for microglia in the neuroinflammation that accompanies trauma and neurodegeneration [16–18]. Without question microglia, the brain's resident macrophages, are vital players in early development and in the innate immune response within the brain. However, our focus here is on the interplay between the systemic innate immune response to injury and possible mechanisms that implicate endothelial cells (ECs) of the BBB as both* target and source* of inflammatory reactions within the brain that promote, amplify, and sustain neuroinflammation that progresses to degeneration.

### 1.2. “Danger” or “Damage” Theory

Theorized by Matzinger in the late 1990s [19], the “danger” or “damage” theory of immunity challenged the dominant self/non-self-basis of immunology. It is based on danger or “alarm” signals that come from the body's own cells and began gaining acceptance in the early 2000s with publication of the EMBO Workshop on Innate Danger Signals and HMGB1 held in February 2006 in Milan [20], although not without vigorous opposition. The key point of the danger theory, in contrast to self/non-self-discrimination, is that self-constituents can also trigger an immune response, if they become damaged or are “dangerous.” This is fundamental to our understanding of how the peripheral innate immune system might activate the ECs of the BBB to orchestrate neuroinflammation that eventually becomes unregulated and uncontrolled.

### 1.3. Alzheimer's Disease (AD)

AD is a chronic neurodegenerative disease responsible for 60 to 70% of cases of all dementia [21–23]. In 2015, approximately 48 million cases of AD were diagnosed worldwide, according to the World Health Organization. The Alzheimer's Association states that about 5 million Americans currently live with AD, and this number is projected and expected to reach about 13.5 million by 2050 [24]. According to the Centers for Disease Control (CDC), in the US, AD is the sixth leading cause of death killing about 94,000 people annually. The annual costs of care to the US are projected to rise from $226 billion in 2016 to $1.1 trillion by 2050, with Medicare and Medicaid paying 70 percent of these costs. Consequently, early, preclinical diagnosis and developing new therapeutic targets to delay AD onset by only five years by 2025 could save an estimated $935 billion over the following 10 years.

Typically beginning in people over the age of 65, late-onset AD (LOAD), the early clinical indicators of AD may include memory loss worsening over time, behavioral signs such as extreme or rapid swings in mood, judgment or disorientation deficits, and problems with language. These initial symptoms are often mistaken for normal aging, further delaying proper diagnosis of AD. Ultimately, bodily functions are progressively lost, leading to death usually by pneumonia. The average life expectancy for an LOAD patient following diagnosis is approximately between 3 and 9 years. In addition to LOAD, accounting for 95% of patients, several genetic mutations exist that cause early onset or familial AD (fAD). Whether LOAD or genetic early onset fAD, neuropathologic hallmarks are extracellular amyloid and neuritic plaques and intracellular neurofibrillary tangle (NFT) formation.

Amyloid beta (A*β*) is the principal constituent of plaques, and soluble levels increase in the blood, in both AD patients and transgenic mouse models, early in the disease [25–29]. Transgenic mice have been generated which mimic some of the features of AD based on amyloid precursor protein, tau, both, or other mutations. In the brain A*β* aggregates promote a chronic neuroinflammatory response mediated by activated microglia and astrocytes and microvascular ECs [30–32]: amyloid plaques → NFTs → neuroinflammation. Beginning in 1992 this had prompted creation of the amyloid metabolic cascade hypothesis [33] and since then much debate has ensued with some continuing pros [34, 35] but many more cons [34, 36–38] after it has been critically reexamined. Unfortunately, since LOAD is not associated with genetic mutations information from transgenic animal models cannot be fully extrapolated to the bulk of human AD pathology. Furthermore, microglial activation and other aspects of parenchymal neuroinflammation along with oxidative stress—reactive oxygen species (ROS) and nitric oxide (NO) formation—can actually precede neuronal damage [39–41] prior to AD histopathologic lesions.

Consequently, the pathogenesis of AD remains poorly understood although major risks to develop the disease are believed to be genetic, even for LOAD, and this includes alleles of apolipoprotein E [42, 43]. However, other nongenetic or epigenetic risk factors may be as or more significant for the 95% LOAD patients especially traumatic brain injury (TBI) but also hypertension, type 2 diabetes mellitus, and a number of modifiable factors including smoking [44]. Because of the failure of randomized clinical trials (RCTs) based on the amyloid hypothesis and using recruitment of AD patients with established symptoms, recent emphasis has been placed by a number of panels and working groups on developing tests to diagnose AD prior to symptom development [45]. Unfortunately, a number of current standard tests are quite invasive, expensive, and poorly tolerated including the sampling and analysis of cerebrospinal fluid for *β*-amyloid or tau proteins [46]. In addition to diagnostic screening of at-risk populations prior to symptom development blood-based biomarkers can also be useful for detecting and monitoring efficacy of therapeutic candidates on the disease progression and as safety markers to detect and monitor potential side effects of drug candidates at the earliest time possible. Therefore, the discovery of equally effective and highly predictive blood-biomarkers is now becoming a major priority in AD therapeutic trial research. Antecedent TBI may be particularly appealing for biomarker screening studies since numerous studies identify it as the most prominent nongenetic risk factor for LOAD development [47].

### 1.4. Parkinson's Disease (PD)

In addition to AD, PD is also intimately associated with neuroinflammation, together with its neuropathologic hallmarks of Lewy bodies (LBs) in dopaminergic (DA) neurons and their degeneration in the* substantia nigra pars compacta* (SNpc) [7, 48]. Similar to AD, PD is a slowly evolving, long-term neurodegeneration of the CNS but one affecting primarily motor pathways [49–51]. PD, also known as the “shaking palsy,” consists of signs that include shaking (tremor), rigidity, and slowness of movement (bradykinesia), which generally become manifest over time. Dementia may appear late in the advanced stages of PD along with anxiety and depression, although in significant numbers these neuropsychiatric manifestations may actually precede motor signs. As with AD the pathogenesis of PD is unknown but like AD genetics plays a significant role in a number of cases. Other factors that may play prodromal roles in its development include TBI and exposure to certain pesticides. In contrast to AD, nicotine contained in tobacco smoke seems to have a beneficial and protective effect.

The most evident symptoms of PD affecting motor functions are the results of the cell death of DA neurons in the midbrain SNpc. The causes of neuronal cell death in the SNpc are not well understood but appear to involve the accumulation of aggregated proteins such as *α*-synuclein into LBs within these neurons [49, 51]. Recent data show that over 50 million cases of PD were diagnosed globally with a death toll of over 100,000 worldwide. As with AD, there is no effective cure or treatment to halt progression but symptomatic treatments with L-Dopa and DA agonists or deep brain stimulation exist to help restore motor functionality in individuals whose quality of life has been greatly impaired by disease progression. Diagnosis of PD is primarily based on signs and symptom presentation with the aid of neuroimaging techniques to rule out other possible disorders.

Again, the focus of many studies and reviews is parenchymal neuroinflammation, wherein microglia and astrocytes lead to progressive death of SNpc DA neurons [52]. In this regard, microglia are viewed as initiating inflammatory responses with slower responding astrocytes amplifying them [53]. Important to these studies, given the availability of transgenic models, are the roles of PD-associated genes and neuroinflammation [54]. These include not only the *α*-synuclein gene but also* parkin*, mutations which are the most common cause of recessively inherited PD [52]. In this regard, variations in another gene, the leucine-rich repeat kinase 2 (LRRK2) gene, have been found in both familial and sporadic PD, which appears to play crucial roles in peripheral inflammation, since LRRK2 is abundant in piWBC. Studies show that giving the prototypic PAMP, LPS, to LRRK2 mutant mice increases cytokine production in their microglia compared to wild-type (WT) mice [52].

As already discussed, innate immune signaling from the periphery to the brain is usually transient, and no evidence exists that this leads to permanent brain tissue damage. However, when signaling is prolonged then parenchymal neuroinflammatory changes become obvious. Just what underlies the mechanisms for this prolongation is the critical question. Perhaps less studied in this regard is the role transmigration of piWBC across the BBB in neuroinflammation plays with neurodegenerative diseases [55]. As with AD and other neurodegenerative diseases, close interplay of the systemic immune system and PD progression is known. Crosstalk between underlying molecular mechanisms of sepsis and SIRS is likely to lead to better understanding of the CNS and innate immune system relationship that should help to clarify PD pathogenesis. Important here is that like AD systemic infection may contribute to PD progression and even its etiology [52].

### 1.5. Amyotrophic Lateral Sclerosis (ALS)

ALS, also known as* Lou Gehrig's or motor neuron disease*, is a neurodegenerative disorder characterized by a progressive loss of control of voluntary movements and muscle weakness and atrophy of extremity and trunk skeletal muscles, as well as muscles of the neck, face, and tongue. This is caused by the degeneration of upper and lower motor neurons in the spinal cord and brainstem [56, 57]. ALS patients may experience (depending on the stage of the disease) muscle stiffness, pain, and atrophy with progressive weakness that ultimately may impair speaking, swallowing, and eventually breathing. The pathogenesis of ALS is unknown in the vast majority of cases, sporadic (sALS), with only a small minority attributed to genetic mutations, fALS [58]. The typical age of onset is in the 50s while much younger onsets are also seen especially in familial (fALS) cases [59].

As in AD and PD ample evidence exists for neuroinflammation and peripheral inflammation in both sALS and fALS [60–62]. More than 25 years ago piWBC were identified within the spinal cords of ALS patients [63]. Based on this and antibodies found Appel and colleagues initially implicated autoimmunity in ALS pathogenesis [64–66]. Autoimmunity is less recognized today but mention has already been made of TLRs and the advanced glycation end products receptor (RAGE [11, 67] in innate immunity and these have been found to be increased in spinal cords of ALS patients and in SOD1 transgenic mice, as reviewed [68, 69]. Subsequent efforts have focused on both reactive ROS and the innate immune system, which appears inextricably linked to this devastating disease [68, 70–72]. One proposed mechanism of ALS (which incorporates genetic mutations of RNA binding proteins, mitochondrial disfunction, and ROS/inflammation) suggests that over time the ability of the cells to be safeguarded against the genetic mutation due to increasing ROS and resulting inflammation is significantly decreased [73, 74]. Either due to an inability to fully neutralize ROS (which results in oxidative DNA damage) and/or due to impaired mitochondrial function [75], the end result is the death of the most sensitive cells such as neurons, especially motor neurons. Like other neurodegenerative disorders no treatment currently exists to cure or halt progression of ALS. Current pharmacological therapies aim at reducing symptoms and improve both live span and the quality of life of the patients. Also, as in other neurodegenerative diseases, the earliest detection prior to onset of symptoms is the key to accomplish these goals.

### 1.6. Chronic Traumatic Encephalopathy (CTE)

CTE is a progressive neurodegenerative disease found most commonly in subjects (generally athletes practicing contact sports) with a history of repetitive TBIs resulting from either symptomatic or asymptomatic (subconcussive) hits to the head. Martland first described a dementia syndrome in former boxers that often was accompanied by parkinsonian and cerebellar motor signs and which was initially called the “punch-drunk” syndrome [76]. Then beginning in 2005 Omalu and colleagues began reporting neuropathologic findings in former US professional football players [77, 78]. CTE symptoms generally appear 8 to 10 years after cessation of repeat bouts of mild TBI [79]. Initial symptoms include disorientation, disattention, dizziness, and frequent headaches, usually migrainous in type. As the disease progresses, additional symptoms become apparent emphasizing erratic behavior and emotional instability and including memory loss. During the later stages of the disease patients become affected by progressive slowing and parkinsonian muscular movements, tremors, worsening dementia, speech impairment (dysarthria), and difficulty in* swallowing*. Currently clinical diagnosis is difficult and diagnosis is dependent on postmortem neuropathologic examination. As with other neurodegenerations there is no effective treatment available for CTE. Neuropathologically, like AD CTE is a* tauopathy* although criteria have recently been established which distinguish it from AD and other tauopathies in brain tissue, inasmuch as NFTs have been observed in perivascular epicentres in the frontal neocortex whereas in the most severe cases they affect widespread brain regions [80].

To date more than 150 brains have been examined at the Boston University CTE Center and a consensus has been developed by Dr. McKee and other neuropathologists on the criteria for CTE diagnosis [81]. As a part of this a distinctive pattern of perivascular phosphorylated tau distinguished CTE from other tauopathies. In addition to the NFTs, in specific areas as per the consensus report, widespread neuroinflammation exists [1, 82, 83]. As with other neurodegenerations, the lack of distinct biomarkers for CTE is a major roadblock to be overcome for the development of effective preclinical screening tests and prognostic assessments of CTE following TBI.

### 1.7. BBB Dysfunction and Neurodegeneration

The term BBB refers to a dynamic functional interface between the blood circulation and the neural tissue in the CNS which protects the brain (long considered an immunologically privileged site due to the existence of the BBB) from harm and maintains the brain's homeostasis through a tight regulation of what comes in and out of the brain's environment. Originally depicted as a standalone specialized multicellular structure formed by brain microvascular ECs connected by tight junctions, a thick basement membrane, and juxtaposed* astrocytic* endfeet, the BBB has now become an integral part of a more complex biological system known at the neurovascular unit which represents a more elaborate and encompassing structure beyond the historical core BBB. In fact, neurons, microglia, and pericytes are members of the neurovascular unit since they interact with core elements of the BBB and its microvascular components leading to functional interplay of central and peripheral cells (including immune leukocytes) which influence and modulate the barrier functions and its physiological responses. These include pathophysiological conditions such as CNS and peripheral inflammation. As such the brain's status as an immune privileged organ is being reexamined and “BBB dysfunction” (essentially a universal feature associated with animal models of preclinical TBI [84] and a critical characteristic of neuroinflammation) can now be extended beyond the tissue or cellular pathophysiology of the BBB components to encompass the entire neurovascular unit [85–89].

Beyond microglial and astrocytic activation, although not as well appreciated, are the activation and transmigration of blood-borne and activated circulating immune leukocytic cells (piWBC) into the CNS in AD, PD, ALS, and other neurodegenerative disorders all associated with robust neuroinflammation [16, 90–95]. Although inflammatory responses in neurodegenerative diseases denote both glial activation and piWBC transmigration, the relationship between these two different inflammatory pathways is clearly far from being understood. What appears to be critical in both, however, is the dysfunction of the BBB/neurovascular unit system. Appreciated in AD, PD, and ALS [96–98], this is also becoming more recognized in the context of CTE as well [99], and, in this regard, BBB breach may persist for years after TBI [100], which consequently negatively impacts the entire neurovascular unit. However, the precise factors governing the initial disruption of the BBB following TBI that lead to neurodegeneration have not fully been identified.

### 1.8. DAMPs and Coagulation Molecules

Central molecules of the two most potent host defense systems that form a* nexus* at the crossroads of innate immunity are high mobility group box protein 1 (HMGB1) and thrombin. HMGB1 is a nonhistone nuclear protein with dual functions: within cells, it is localized primarily to the nucleus where it binds and bends DNA and plays a role in transcriptional regulation [101]. Once outside the cell it can serve as a proinflammatory cytokine and as a late mediator of sepsis [102]. Beyond infections, HMGB1 has roles during trauma and sterile inflammation, such as in SIRS, where it orchestrates key events including piWBC recruitment and induction to secrete inflammatory cytokines [103, 104]. In addition, once outside cells, HMGB1, also known as* amphotericin*, promotes motility of cells as well as axonal nerve growth and has been found to be essential for brain development [105]. Increasingly, HMGB1, DAMPs, and the “danger” hypothesis are being explored in the CNS and its disorders [105].

### 1.9. HMGB1 and BBB Dysfunction

HMGB1 is released by innate immune cells in response to bacterial LPS or by endogenous TNF and other proinflammatory cytokines from innate immune cells. Externally located HMGB1 binds to PRRs such as TLR2 and TLR4 [11, 106] as well as RAGE [107, 108]. Evidence indicates that engagement of TLRs is needed for further cytokine production and release while activation of RAGE by HMGB1 induces piWBC recruitment [108]. Of interest, earlier studies indicated that RAGE was also required for neurite outgrowth by* amphotericin* in the developing nervous system [105, 109].

Increased circulating HMGB1 from peripheral systemic inflammation can activate one or more of its receptors such as TLR2 or TLR4 or RAGE on microvascular ECs [110], in a* target-based* approach. Based on recent published evidence, a mechanism by which HMGB1 can influence piWBC recruitment is by its formation of a heterocomplex with the homeostatic chemokine CXCL12 on these cells [111]. This heterocomplex appears to act more potently on the CXCR4 receptor than on CXCL12 alone and CXCR4 expression on ECs of the BBB has been shown to enhance transmigration of piWBC [112]. Thus, the* HMGB1-CXCL12-CXCR4* axis may represent a new “player” in the transEC migration of piWBC in BBB/neuroinflammation leading to neurodegeneration.

### 1.10. Coagulation Cascade: Thrombin and BBB Dysfunction

Thrombin is the proinflammatory serine protease essential as the ultimate protease in the coagulation pathway, and as prothrombin it circulates at micromolar concentrations. By activation of a small family of G-protein-coupled receptors, known as PARs (proteinase-activated receptors) [113], thrombin has been found to have extensive roles within developing nervous system and following injury or degeneration outside of its pivotal position in coagulation [114, 115]. We postulated that both HMGB1 and thrombin may play a significant role in BBB disruption since both are proinflammatory and both are known to disrupt vascular barriers in other tissues [110, 116–121].

In an attempt to explain thrombin's effect on brain edema Guan and colleagues injected thrombin stereotactically into rat caudate nuclei and found extravasation of Evans Blue dye [122]. They also found that in addition adding thrombin to EC cell cultures increased expression of matrix metalloproteinase-2, which was proposed to occur by activating PAR1. In similar experiments, Garcia's group and others showed thrombin-mediated disruption of several microvascular barriers via a PAR1 mechanism [110, 116–121]. In a recent study by Festoff et al., both thrombin and HMGB1 can directly impair BBB integrity in vitro [123].

Over the last decade it has become increasingly appreciated that inflammation and coagulation are linked evolutionary defense systems [124, 125], a fact that is slowly becoming recognized in the CNS as well. In this regard, TBI, ischemic and hemorrhagic stroke are characterized by increased levels of intraparenchymal thrombin and HMGB1 as well as BBB dysfunction [126, 127]. In the brain cell low concentrations of thrombin act through its principal receptor, PAR1, to induce neuroprotection [115]. In contrast, at higher concentrations thrombin causes brain damage [128] where it appears to act via PAR4 [129–131]. Thrombin directly affects the activity of multiple cell types and regulates a variety of biological functions, including inflammation, leukocyte migration, and vascular permeability through PAR activation [132–135]. Furthermore, direct links also exist between thrombin and HMGB1: HMGB1 is involved in a number of systemic vascular diseases [136, 137] and is also increased in stroke [105, 138], while both HMGB1 and thrombin are released in various neurologic conditions and HMGB1 promotes coagulation [139]. Of interest, in TBI, the critical nongenetic antecedent event in AD, PD, and CTE, HMGB1 and thrombin—post-TBI coagulopathy [140, 141]—are both increased. Taken together, these observations raise the possibility that HMGB1 and thrombin participate during neuroinflammatory situations such as occurs post-TBI/CTE as well as in AD, PD, and ALS, which contribute to BBB dysfunction and transendothelial migration of piWBC.

One particular linkage topic that relates to the BBB as potentially revealing new therapeutic targets in AD is A*β* transport in and out of the brain. A number of reports have emphasized RAGE and the low density receptor related protein (LRP-1) in this capacity [26, 86]. Most consider that RAGE is the primary transporter of A*β* from blood to brain, while LRP-1 mediates transport of A*β* the opposite way. Consequently, a therapeutic approach might focus on interrupting RAGE binding to A*β*, and an oral, small-molecule inhibitor of RAGE, Azeliragon (TTP488), for mild AD entered Phase 3 trials in the US and Canada in 2015 (STEADFAST).

Furthermore, HMGB1 is quite susceptible to changes in redox state, both ROS and NO. Within the nucleus HMGB1 contains two DNA-binding HMG box domains (N-terminal A and central B). Recent evidence indicates that HMGB1 also normally translocates to the mitochondrion, where it affects mitochondrial quality control [142]. In normal brain cells the cysteines of the A-box (Cys23, Cys45) and B-box (Cys106) are reduced (-SH) allowing HMGB1 to bind DNA and translocate to/enter mitochondria. Reduced, nuclear, and mitochondrial HMGB1 can be actively secreted from macrophages and dendritic cells [142, 143]. In addition, HMGB1 can be released from* exploding* necrotic cells, while typically apoptotic cells retain HMGB1, which is tightly attached to hypoacetylated chromatin. Because of this, HMGB1 is not usually released from apoptotic cells and does not induce inflammation [144]. In neuroinflammation following TBI and stroke or in neurodegeneration, the damaged neural cell becomes oxidized and disulfide (S-S) bridges are formed between Cys23 and Cys45 while Cys106 can remain -SH; in this situation HMGB1 is proinflammatory. If it becomes completely oxidized; however, an additional S-S is formed with Cys23 and Cys45 and now HMGB1, as* amphotericin*, can promote regeneration; that is, it stimulates nerve growth [109, 145]. HMGB1 and TBI are actively being investigated including increased brain expression [146] and plasma levels associated with outcome after injury [147]. The relationship between HMGB1 and mitochondria, perhaps the HMGB1 fraction translocated to these organelles, is being established. HMGB1 and other DAMPs such as mitochondrial DNA [148], and other mitochondrial DAMPs released from mitochondria by trauma and other stimuli [9], can figure critically in development of neuroinflammation, as in systemic inflammation [10], leading to neurodegeneration.

Outside of the cell oxidized HMGB1 is known to ligate three different PRRs, all of which are expressed on the surface of cerebrovascular ECs. These include TLR2 and TLR4 [149–151] as well as RAGE [107, 152, 153]. Each of these PRRs binds a variety of ligands, besides HMGB1, most of which are critical in determining vascular complications of different diseases such as diabetes and atherosclerosis [67, 154]. Both TLR and RAGE ligation leads to NF*κ*B activation that is sustained, and this in turn increases PRR expression, as well as TNF production [107]. This ensures that the inflammatory signal is maintained and amplified [67, 154]. Signal transduction through TLRs involves the Toll/IL-1 receptor (TIR) domain (TIR) [155] and has both MyD88-dependent and independent pathways. MyD88 is essential for induction of inflammatory cytokines triggered by all TLRs, while a MyD88-independent pathway is specific for TLR4 and TLR3.

Thrombin has also been associated with ROS and, in particular, ROS-mediated membrane lipid peroxidation (MLP) [156, 157]. Prothrombin, like TM, is produced by astrocytes in normal brain [158] and both thrombin and prothrombin have been found to be associated with plaques and NFTs in AD brains [159]. We have recently shown that MLP is critical in neurodegeneration, both AD and PD, and involves thrombin and PAR1 [160]. In turn, ROS is clearly increased in association with neurodegeneration [161]. Furthermore, in support of the BBB in AD as source of proinflammatory factors, reports have shown an increase in thrombin and other proinflammatory factors in AD ECs [162].

Consequently, inhibiting RAGE might also produce beneficial results apart from A*β* transport by interrupting HMGB1 signaling through this receptor. Such activity would be additive in this context since HMGB1 also binds to and signals via TLR2 and TLR4 as well [106]. Recent interpretations suggest that HMGB1-RAGE is instrumental for piWBC infiltration [104, 111], whereas HMGB1-TLR4 may be responsible for cytokine production [11, 163].

### 1.11. Blood Markers of Microvascular Damage

The chondroitin sulfate proteoglycan, thrombomodulin (TM), is ubiquitously present on the surface of ECs [125]. We also found TM on mouse astrocyte surfaces [164] where it was functionally active and similar to the EC molecule. TM is an endogenous anticoagulant, one of three natural anticoagulant mechanisms, since it binds thrombin with high affinity and also the circulating zymogen protein C (PC) to form activated PC (APC) which then inactivates factors Va and VIIIa to stop coagulation [13, 125]. Beyond braking clotting, APC can also dampen inflammation, which it accomplishes in several ways: (1) by inhibiting expression of tissue factor (TF) and release of proinflammatory cytokines by monocytes; (2) by blocking expression of leukocyte adhesion molecules; and (3) by inhibiting neutrophil chemotaxis and cytoprotection [13, 125]. In this regard, recombinant TM (rTM) is cytoprotective since it binds thrombin, preventing its activation of PARs on neural or immune cells [165, 166]. We found it enhanced recovery after spinal cord injury in rats [167] and proposed at the time that the most attractive mechanism was binding of thrombin by rTM preventing its activation of specific PARs. However, using a slightly different rTM others found similar results that they attributed to APC's effects on activation and inhibition of leukocyte migration [168], as reviewed [169]. Important to note here is that thrombin is a potent inducer of microvessel hyperpermeability that is mediated by Rho kinase-dependent myosin light chain-2 phosphorylation and Ca^2+^ Influx through the Na^+^/Ca^2+^ exchanger. These interactions ultimately activate the contractile mechanism of the endothelium leading to the physical opening of the interendothelial clefts and loss of BBB integrity [170, 171]. Furthermore, APC possesses various cytoprotective functions which, in addition to antiapoptotic and anti-inflammatory activities, include endothelial barrier stabilization. These cytoprotective activities seem to require both the endothelial protein C receptor (EPCR) and a subpopulation of PAR1, whereas APC elicits cytoprotective signaling through cleavage of these atypical PAR1 receptors leading to the activation of Rac1 signaling which promotes endothelial barrier protective responses [172, 173].

### 1.12. TM: Multifunction or Do-All Receptor at the BBB

**Figure 1:**
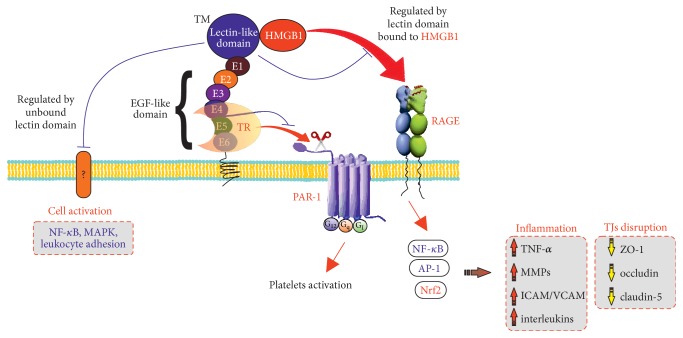
TM as a multifunctional or “Do-All” receptor, after Esmon [13] in considering roles at nexus of coagulation and innate immune inflammation at the BBB.

Beyond anticoagulation, TM also functions as a natural anti-inflammatory agent [174] that has been attributed not to its thrombin and PC binding domain (known as TMD2/3) but to its NH2 terminal C-type lectin-like domain (TMD1) [125, 175, 176]. A separate explanation for the anti-inflammatory effects of rTM, apart from APC generation to inactivate Factors V and VIII, came from studies showing that TMD1 binds HMGB1 very tightly [177]. This path-finding study, and subsequent other novel ones in which the D1 domain was “knocked in” to produce transgenic mice lacking this C-type lectin-like domain (called TM^LeD/LeD^ mice) [178], suggested that more than one anti-inflammatory mechanism might account for TM's effects [179]. Mechanistically, TMD1 binding to HMGB1 would prevent its engagement of RAGE [180] and/or TLR2/TLR4 [106]. Because of this Esmon [13] considered TM a “do-all” receptor bridging the nexus—the crossroads—of coagulation and inflammation (innate immunity). His schematic for TM showing functions discussed above is shown in Figure 1.

However, TM's anti-inflammatory mechanism may be more complicated since, in addition to HMGB1, TMD1 also actively binds to the carbohydrate Lewis Y (Le^y^) antigen in LPS [175]. By binding to the Le^y^ antigen, rTMD1 is able to block the interaction of LPS with CD14 and/or TLRs, reducing subsequent LPS-induced inflammatory reactions and thereby suppressing downstream inflammatory signaling [176]. Consequently, in addition to thrombin binding and APC activation TM provides anti-inflammatory regulation via TMD1 binding of both HMGB1 and the Le^y^ antigen.

Considerable evidence exists that blood levels of soluble TM (sTM) and von Willebrand factor (vWf) can serve as surrogate markers for microvascular damage [181, 182]. Although few in number, several studies have also evaluated the plasma levels of sTM levels in different CNS diseases such as AD [183] and multiple sclerosis (MS) patients [184] suggesting that sTM is potentially a good marker to assess brain (BBB) microvascular EC damage.

More recently, in our efforts to develop a validated marker for conversion of MCI to AD we measured both sTM antigen (TM-Ag) and a functional assay (TMa) [185, 186] for TM activation of PC to APC. We found significant increases above age-matched controls when MCI and AD levels are grouped. However, MCI sTM levels were, in fact, greater than in AD patients. But when TM-Ag was analyzed specifically the following relationship was found: AD > MCI > control [123]. In addition to thrombin, the prototypic DAMP alarmin, HMGB1, dramatically enhanced in vitro BBB permeability to several molecular weight dextran whether at 3 or 6 hr incubations at ng/mL concentrations. Others have shown that in rats whether BBB dysfunction was due to experimental stroke or TBI, a neutralizing monoclonal antibody (mAb) to HMGB1 prevented the BBB dysfunction [126, 127]. This same group has shown more recently that anti-HMGB1 mAb provides neuroprotection in a rat model of PD by attenuating the BBB breach in this disease [187]. We directly correlated such direct effects with levels of several of these molecules present in sera from AD, MCI, and control samples and we found direct correlation with both sRAGE and HMGB1 with A*β* [123].

**Figure 2:**
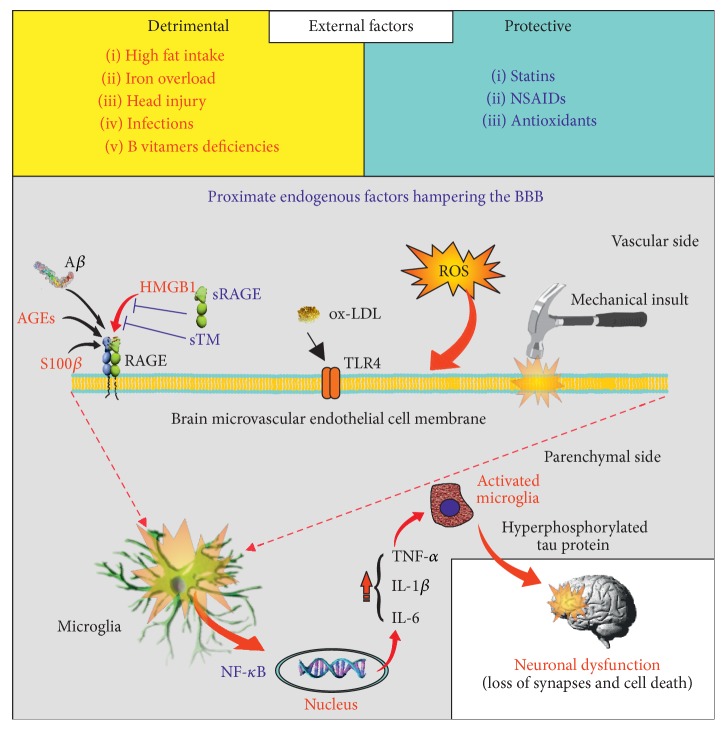
Schematic based on data, both ours and others, where proximate factors generated from external sources, including dietary, infections, and injury, act on brain microvascular ECs to cause a “breach” of the BBB/NVU. The proximate factors include molecules associated with innate immune activation (DAMPs and A*β*) while agents that can interdict these factors include sTM and sRAGE. The initial and subsequent episodes of the dysfunction can “fan the flames” of neuroinflammation within the brain with microglial (and astrocytic) activation at transcriptional levels resulting in mitochondrial dysfunction, tau hyperphosphorylation, and aggregation, synapse loss, and neuronal cell death.

Figure 2 represents our current concepts as to how* coag-inflammatory* molecules such as DAMPs, thrombin, and TM might interact with CNS injury, cytokines, and other proinflammatory molecules and A*β* in the breach of the BBB/neurovascular unit leading to neuroinflammation and, ultimately, neurodegeneration. Incorporated within this concept is how we might use this information to develop relatively noninvasive blood-based biomarkers to diagnose these conditions* before* the onset of symptoms or signs and to develop new therapeutic targets to prevent evolution of these disorders after initial injuries develop.

## 2. Conclusions

The BBB and greater neurovascular unit might function as both* source* and* target* of inflammatory factors since over the last 15 years studies have shown that the cerebral microcirculation is in an “activated proinflammatory” state in neurodegenerative diseases such as AD [188]. One such target may be TM since we found that as with relapsing remitting MS patients [184] increased levels of sTM occur in sera of AD and MCI patients compared with controls [123], clearly a source of this anti-inflammatory/anticoagulant. In turn, when HMGB1 binds to* target* RAGE and/or TLRs on brain microvascular ECs they are* source* of proinflammatory agents by releasing TNF and other cytokines [188].

An increased understanding of the role of HMGB1 and other DAMPs, along with thrombin/PARs in the activation and transendothelial migration of piWBC contributing to neuroinflammation in AD, PD, and all neurodegenerative diseases as well as neurotrauma, may allow discovery of novel therapeutic targets and treatment strategies. Not only might these facilitate treatment to halt progression in these poorly treated and currently not curable diseases, but also they might aid in detecting the conversion from minimal deficit or preclinical condition to disease in other neurological disorders that display BBB dysfunction that lead to the migration of inflammatory cells into the CNS. Noteworthy to mention here is also the fact that inflammatory diseases of the gastrointestinal tract such as intestinal inflammatory bowel diseases, which includes Crohn's disease and ulcerative colitis, can affect the CNS leading to behavioral symptoms and cognitive dysfunction [189]. This further emphasizes the potential impact of peripheral inflammation on the CNS.

## Abbreviations

ALS: Amyotrophic lateral sclerosis
AD: Alzheimer's disease
A*β*: Amyloid beta
APC: Activated PC
BBB: Blood-brain barrier
CTE: Chronic traumatic encephalopathy
DAMPs: Damage-associated molecular patterns
EC: Endothelial cell
HMGB1: High mobility group box protein 1
LBs: Lewy bodies
LPS: Lipopolysaccharide
LRP-1: Low density receptor related protein
LRRK2: Leucine-rich repeat kinase 2
MCI: Mild cognitive impairment
MLP: Membrane lipid peroxidation
MS: Multiple sclerosis
NFT: Neurofibrillary tangles
PAR: Protease activated receptor
PD: Parkinson's disease
PRRs: Pattern recognition receptors
SIRS: Systemic inflammatory response syndrome
SNpc: Substantia nigra pars compacta
sRAGE: Soluble receptor for advanced glycation end products
TBI: Traumatic brain injury
TLR: Toll-like receptor
TM: Thrombomodulin
TNF: Tumor necrosis factor
WBC: White blood cells.
